# Functional MAIT Cells Are Associated With Reduced Simian–Human Immunodeficiency Virus Infection

**DOI:** 10.3389/fimmu.2019.03053

**Published:** 2020-01-17

**Authors:** Amudhan Murugesan, Chris Ibegbu, Tiffany M. Styles, Andrew T. Jones, Uma Shanmugasundaram, Pradeep B. J. Reddy, Sadia J. Rahman, Piu Saha, Matam Vijay-Kumar, Esaki Muthu Shankar, Rama Rao Amara, Vijayakumar Velu

**Affiliations:** ^1^Emory Vaccine Center, Emory University, Atlanta, GA, United States; ^2^Department of Microbiology and Immunology, Yerkes National Primate Research Center, Emory University, Atlanta, GA, United States; ^3^Department of Physiology and Pharmacology, University of Toledo College of Medicine and Life Sciences, Toledo, OH, United States; ^4^Department of Life Sciences, Central University of Tamil Nadu, Thiruvarur, India

**Keywords:** MAIT cells, HIV/SIV infection, CD8 T cells, rhesus macaques, innate like T cells, microbial metabolites, MR-1 tetramer, cytokine treatments

## Abstract

Mucosa-associated invariant T (MAIT) cells are recently characterized as a novel subset of innate-like T cells that recognize microbial metabolites as presented by the MHC-1b-related protein MR1. The significance of MAIT cells in anti-bacterial defense is well-understood but not clear in viral infections such as SIV/HIV infection. Here we studied the phenotype, distribution, and function of MAIT cells and their association with plasma viral levels during chronic SHIV infection in rhesus macaques (RM). Two groups of healthy and chronic SHIV-infected macaques were characterized for MAIT cells in blood and mucosal tissues. Similar to human, we found a significant fraction of macaque T cells co-expressing MAIT cell markers CD161 and TCRVα-7.2 that correlated directly with macaque MR1 tetramer. These cells displayed memory phenotype and expressed high levels of IL-18R, CCR6, CD28, and CD95. During chronic infection, the frequency of MAIT cells are enriched in the blood but unaltered in the rectum; both blood and rectal MAIT cells displayed higher proliferative and cytotoxic phenotype post-SHIV infection. The frequency of MAIT cells in blood and rectum correlated inversely with plasma viral RNA levels and correlated directly with total CD4 T cells. MAIT cells respond to microbial products during chronic SHIV infection and correlated positively with serum immunoreactivity to flagellin levels. Tissue distribution analysis of MAIT cells during chronic infection showed significant enrichment in the non-lymphoid tissues (lung, rectum, and liver) compared to lymphoid tissues (spleen and LN), with higher levels of tissue-resident markers CD69 and CD103. Exogenous *in vitro* cytokine treatments during chronic SHIV infection revealed that IL-7 is important for the proliferation of MAIT cells, but IL-12 and IL-18 are important for their cytolytic function. Overall our results demonstrated that MAIT cells are enriched in blood but unaltered in the rectum during chronic SHIV infection, which displayed proliferative and functional phenotype that inversely correlated with SHIV plasma viral RNA levels. Treatment such as combined cytokine treatments could be beneficial for enhancing functional MAIT cells during chronic HIV infection *in vivo*.

## Introduction

MAIT cells are a distinctive innate-like T cell subset with antimicrobial activity against bacteria and yeast that utilize riboflavin metabolism pathway (1). They are defined by the presence of a semi-invariant T cell antigen receptor (TCR) α chain paired with limited TCR Vβ chains (2), and they have a unique expression of invariant T cell receptor Vα7.2-Jα33 in humans and Vα19-Jα33 in mice (2, 3). MAIT cells recognize microbial vitamin B2 metabolites presented in association with evolutionarily conserved MHC class Ib-related molecule MR1 (1). MAIT cells are phenotypically identified as T cells co-expressing TCRVα7.2 and CD161, a lectin receptor (4, 5) in humans. IL-18R is another surrogate marker for MAIT cells and is regulated by transcription factors promyelocytic leukemia zinc finger (PLZF) and RAR-related orphan receptor gamma (ROR-gamma) (2, 6, 7). MAIT cells also express higher levels of chemokine receptor CCR6 (5, 6). They respond through secretion of pro-inflammatory and tissue-protective cytokines like interleukin IL-17, IFN-γ, TNF-α, and IL-22 (7, 8). They are mainly distributed in tissues such as spleen, lymph node, lung, liver, and gut (9–11). These cells are activated either in a MR-1-dependent or -independent manner; viruses activate these cells through the MR-1-independent pathway by cytokines such as IL-18 and IL-12 (4). Previous studies have shown that MAIT cells are effector memory phenotype in humans and express tissue-homing markers such as CCR6, CCR5, and CXCR6 (11, 12). MAIT cells have been implicated in primary immune responses due to their presence at mucosal surfaces (8, 13). Loss of these cells has been associated with pathogenesis of various infectious and non-infectious diseases like HIV, HBV, and HCV (9, 10, 12, 14–17), type II diabetes (18), inflammatory bowel disease (19), and tuberculosis (20–22).

Previous studies in HIV infection have shown that MAIT cells are reduced in periphery. Similarly, MAIT cells have been shown to be reduced systemically in SIV-infected rhesus macaques (23). However, a recent study in SIV/SHIV-infected pigtail macaque, MAIT cells are shown to be increased in the blood (24). Results from these studies vary, and very few studies have been performed in rhesus macaques with SIV/SHIV infection. Hence, we conducted a comprehensive analysis of the MAIT cell population in blood and tissue compartments in uninfected and chronic SHIV1157ipd3N4 (>14 weeks post-infection)-infected RM. To study MAIT cells in rhesus macaque models, we obtained a RM-specific MR1 tetramer loaded with the MAIT cell Ag 5-OP-RU (MR1-5-OP-RU tetramer) from NIH core facility. Using this macaque-specific MR1 tetramer, we assessed the phenotypic similarities between MR1 tetramer vs. human MAIT cell surface markers such as CD161 and TCR7.2. We find that RM MAIT cells are phenotypically similar to human MAIT cells. Our data reveal that there is an enrichment of MAIT cells in the blood during chronic SHIV infection (12). Unlike resting MAIT cells, which are not usually activated (10, 25, 26), MAIT cells during chronic SHIV infection are activated, express higher levels of functional markers such as Ki-67, granzyme-B, and perforin, and maintain IFN-γ production. Unlike blood MAIT cells, rectal MAIT cells are unaltered post-SHIV infection. Interestingly, MAIT cells from blood and rectum are highly proliferative with higher cytotoxic phenotype, which is inversely correlated with plasma viral RNA levels and directly correlated with CD4 T cell levels in both blood and rectum. Since MAIT cells exert their antimicrobial function and help to fight bacterial infection, we stimulated MAIT cells with bacterial products during chronic SHIV infection. MAIT cells responded to microbial stimuli and make cytokines such as IL-17, IFN-γ, TNF-α, and IL-22. Markers of microbial products flagellin and lipopolysaccharides (LPS) displayed increased trend during SHIV infection, yet only immunoreactivity to flagellin correlated positively with MIAT cell frequency in blood. We also looked at the soluble factors (cytokines) that are important for the expansion of functional MAIT cells during chronic SHIV infection *in vitro*. Among the cytokines studied, IL-7 seems to be an important cytokine responsible for the proliferation of MAIT cells. Unlike IL-7, cytokines IL-12, and IL-18 seem to be important for the induction of cytotoxic molecules on MAIT cells. We believe cytokine therapies such as IL-7, IL-12, or IL-18 might be useful in activating functional MAIT cell function *in vivo* during chronic HIV infection.

## Results

### Identification of MAIT Cells Using TCR7.2, CD161, and MR1 Tetramer in SHIV-Naïve Rhesus Macaques

**Figure 1:**
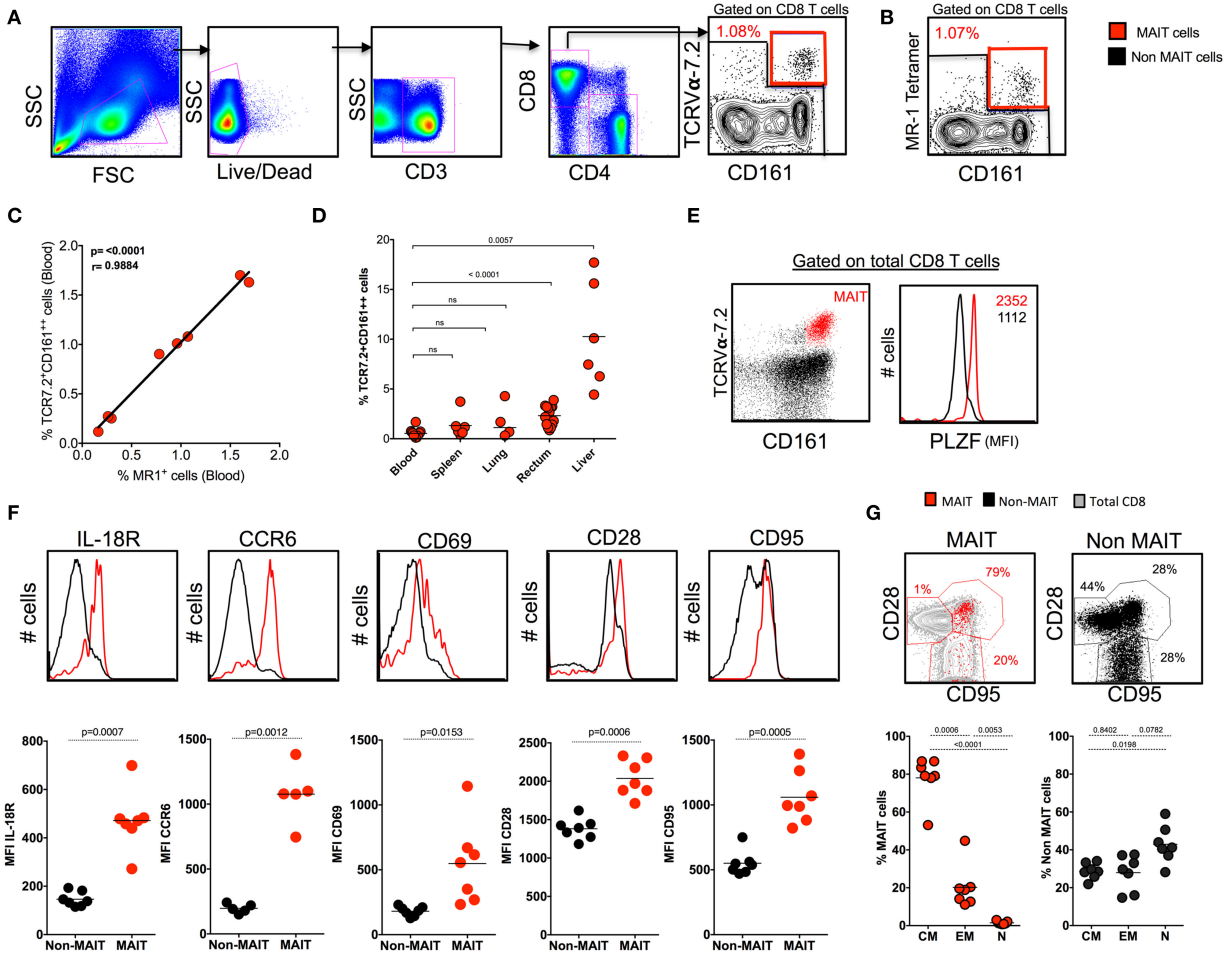
Identification of MAIT cells using TCR7.2, CD161, and MR1 tetramer in SHIV-naïve rhesus macaques. **(A)** Representative FACS plot showing the gating strategy for identifying MAIT cells (CD161^++^ TCR7.2^+^ T cells) in healthy rhesus macaques. MAIT cells are shown in red, and non-MAIT cells are shown in black. **(B)** Representative flow plot shows MAIT cell (gated on CD3^+^CD8^+^ cells) characterized by the expression of mamu MR-1 tetramer (5-A-RU) staining from the same animal displayed in **(A)**. **(C)** Graph showing the correlation between CD161^++^TCR7.2^+^ T cells and MR1^+^CD161^++^ T cells (*n* = 8). **(D)** Dot plot showing the frequency of MAIT cells in various tissues form SHIV-naïve animals. **(E)** Representative flow plots showing the expression of MAIT cells with CD161 and TCR7.2 and its relative expression of transcription factor PLZF. **(F)** Representative flow plots showing the expression of different phenotypic markers IL-18R, CD69, CCR6, CD28, and CD95 by MAIT and non-MAIT cells, mean fluorescence intensity of each marker is displayed. **(G)** Representative FACS plots showing the expression of CD28 and CD95 on MAIT and non-MAIT cells of blood and also the frequencies of central memory (CD28^+^CD95^+^), effector memory (CD95^+^CD28^−^), and naïve (CD28^−^CD95^−^) between MAIT and non-MAIT cells (*n* = 8).

Human studies have identified MAIT cells based on the expression of surface markers CD161 and TCRVα7.2 and confirmed them with MR1 tetramers (12, 27). Similarly, we phenotypically characterized MAIT cells in the blood of SHIV-naïve RM based on the expression of CD3^+^CD8^+^CD161^++^TCR7.2^+^ (Figure 1A) and compared them with the expression of macaque MR1 tetramer (Figure 1B). The frequency of MR1 tetramer positively (*p* < 0.0001, *r* = 0.98) correlated with our CD3^+^CD8^+^CD161^++^TCR7.2^+^ population in RM, suggesting that most (98%) of the CD161^++^TCR7.2^+^ cells identify MAIT cells in SHIV-naïve RM (Figure 1C). Representative flow plots for MR-1 tetramers 5-A-RU and 6-FP are shown in Supplementary Figure 1A. Among CD3+MR-1+ cells, >94% of the cells are CD8+ cells (Supplementary Figure 1B). Next we compared the frequency of MAIT cells between blood and various tissues in SHIV-naïve RM. Naïve RM tended to have lower MAIT cells in blood (~0.53%), spleen (~0.90%), and lung (~1.09%) compared to the rectum (~2.3%, mean) and liver (~9.8%, mean) (Figure 1D). A key feature of the MAIT cell developmental pathway is the expression of PLZF, and cells have been shown to express the transcription factor PLZF in humans and mice (3, 6, 28, 29); thus, we further characterized the macaque CD8^+^CD161^++^TCR7.2^+^ cells for the expression of PLZF. We observed that the majority of macaque blood CD161^++^TCR7.2^+^ CD8^+^ cells express PLZF (Figure 1E). Similar to blood, CD161^++^TCR7.2^+^ CD8^+^ cells (red) from various tissues (spleen, lung, liver, and rectum) expressed higher levels of the PLZF in SHIV-naïve RM (Supplementary Figure 1C) compared to non-MAIT cells (black). Tissue analysis displayed that rectum and liver expressed higher levels of MAIT cells compared blood, spleen, and lung in SHIV-naïve animals (Figure 1D); representative data on MR-1 staining pattern in liver are shown in Supplementary Figure 1D). Next, to understand their phenotype in RM, we stained for markers pertaining to human MAIT cells using various phenotype markers such as IL-18R, CCR6, CD69, CD28, and CD95 in the blood. Consistent with human MAIT cells (2, 3, 30), the macaque MAIT cells express higher levels of IL-18R, CCR6, CD69, CD28, and CD95 compared to non-MAIT cells (Figure 1F). Interestingly, the RM MAIT cells are predominantly of CD28^+^CD95^+^ memory phenotype, unlike the non-MAIT cells (Figure 1G). These data conclusively suggest that macaque MAIT cells can be identified with surface markers such as CD3^+^CD8^+^CD161^++^TCR7.2^+^ cells.

### MAIT Cells Are Polyfunctional and They Predominantly Make IL-17 in SHIV-Naïve Macaques and Respond to Bacterial Products

**Figure 2:**
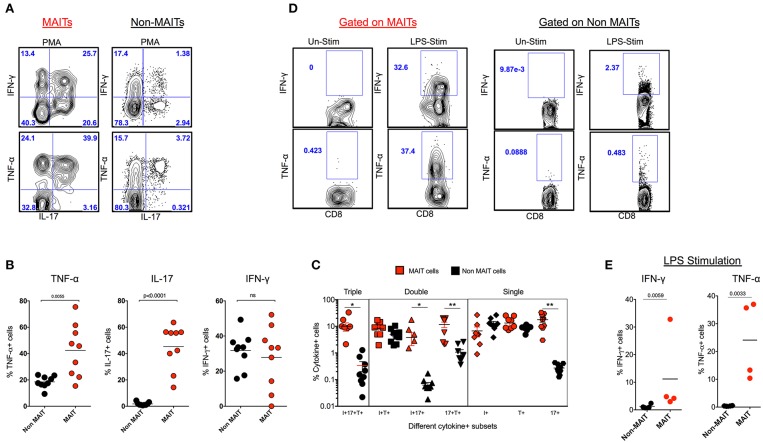
MAIT cells are polyfunctional, and they predominantly make IL-17 in SHIV-naïve rhesus macaques. **(A)** Representative flow plots showing the production of cytokines IFN-γ, TNF-α and IL-17 by MAIT and non MAIT cells from SHIV-naïve animals. **(B)** Cumulative data for the production of cytokines from MAIT (red) and non-MAIT cells (black) from 9 SHIV-naïve animals stimulated with PMA/ionomycin. **(C)** Boolean analysis determining the co-expression pattern of IFN-γ, TNF-α, IL-17 by MAIT and non-MAIT cells. **(D)** Cytokine production capacity of MAIT and non-MAIT cells with LPS stimulation. **(E)** Cumulative data from four animals with LPS stimulation, LPS stimulates cytokine production capacity of MAIT cells. *P < 0.05; **P < 0.01.

In order to understand the cytokine production capacity of MAIT cells in naïve RM, we stimulated total PBMC using PMA/ionomycin and looked for the production of cytokines such as IFN-γ, TNF-α, and IL-17 using standard intracellular cytokine assay (ICCS). Stimulation with PMA/ionomycin triggered the production of IFN-γ, TNF-α, and IL-17, with higher levels of IL-17 and TNF-α and comparable levels of IFN-γ compared to non-MAIT cells (Figure 2A). In order to understand the poly-functionality of MAIT and non-MAIT cells in naïve RM, we performed a Boolean analysis of cytokine-positive cells. Interestingly, MAIT cells are polyfunctional and produce higher levels of triple cytokines (IL-17^+^TNF-α^+^IFN-γ^+^), double cytokines (IL-17^+^TNF-α^+^), (IL-17^+^IFN-γ^+^), and single cytokine-producing cells, predominantly IL-17^+^ cells than non-MAIT cells, suggesting that MAIT cells are predominantly IL-17-producing CD8 T cells (Figures 2B,C). MAIT cells are mostly antibacterial (11, 31, 32); hence, we also looked at the cytokine production capacity of MAIT cells with LPS stimulation in SHIV-naïve RM. We stimulated total PBMC using LPS and looked for the production of cytokines such as IFN-γ and TNF-α, using standard ICCS assay. Stimulation with LPS triggered the production of IFN-γ and TNF-α mainly by MAIT cells. Significantly higher levels of IFN-γ and TNF-α are produced by MAIT cells compared to non-MAIT cells (Figures 2D,E). Collectively, the above data suggest that MAIT cells are polyfunctional cells than non-MAIT cells in SIV-naïve RM.

### MAIT Cells in SHIV-Infected Macaques Are Highly Proliferative and Enriched With Cytotoxic Granules in Blood and Rectum

**Figure 3:**
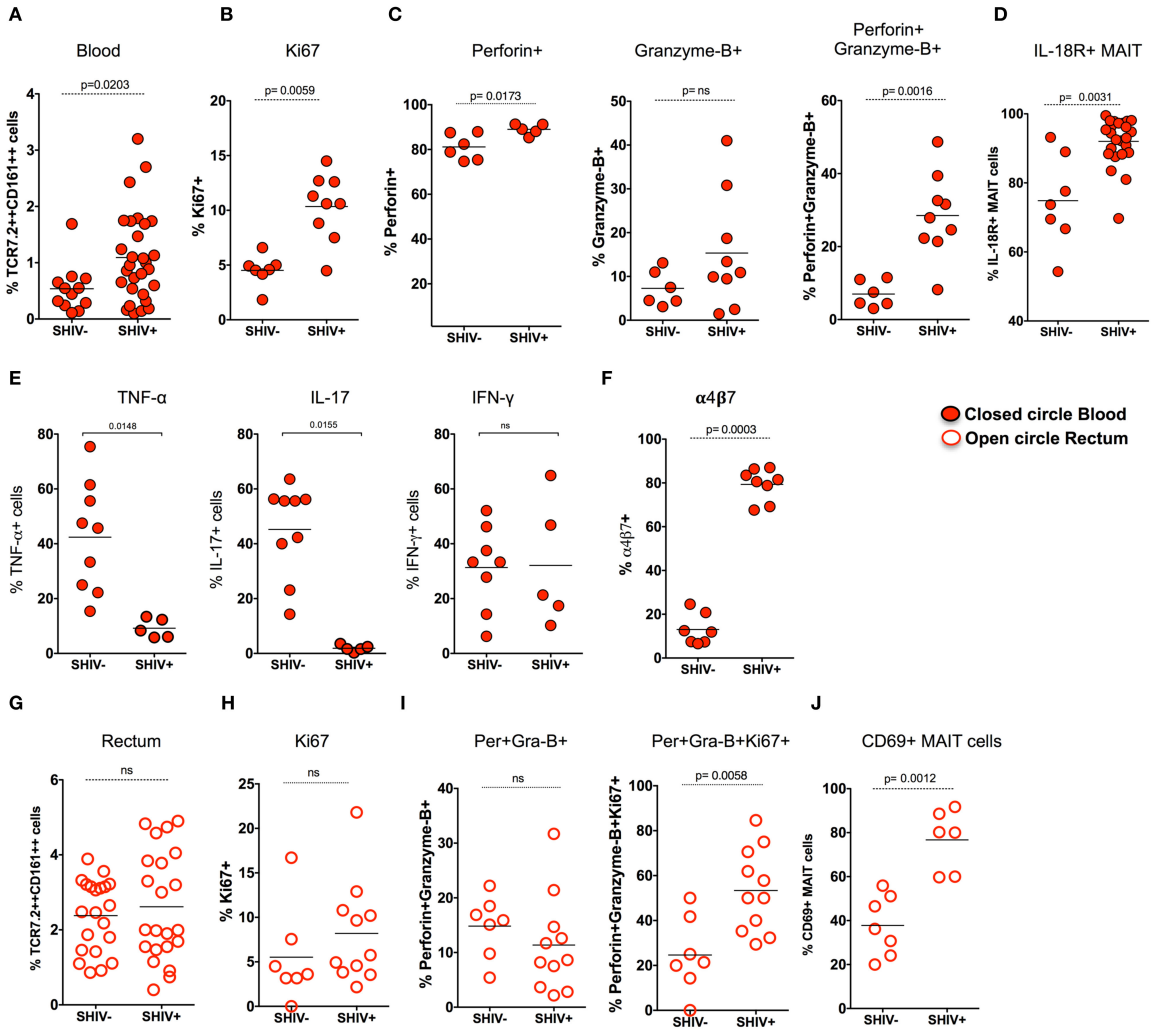
MAIT cells in SHIV-infected macaques are highly proliferative and enriched with cytotoxic granules in blood and rectum. **(A)** Frequency of blood MAIT cells are significantly enriched during chronic (>14 weeks post-SHIV infection (*n* = 29) and SHIV-naïve animals (*n* = 14). **(B)** Frequency of Ki-67+ MAIT cells in blood before and after SHIV infection: SHIV-infected (*n* = 9) and SHIV-naïve animals (*n* = 7). **(C)** Frequency of perforin+, granzyme-B+, and perforin+granzyme-B+ MAIT cells in blood are shown before and after SHIV infection: SHIV infected animals (*n* = 9) and SHIV-naïve animals (*n* = 8). **(D)** Frequency of IL-18R expressing MAIT cells in blood before and after SHIV infection: SHIV-infected (*n* = 21) and SHIV-naïve animals (*n* = 7). **(E)** Plots showing the cytokine-producing ability of MAIT cells post-SHIV infection. MAIT cells predominantly maintain IFN-γ production but lose the capacity to make TNF-α and IL-17 in SHIV-infected animals (*n* = 5) and SHIV-naïve animals (*n* = 9). **(F)** Data for the frequency of α4β7+ MAIT cells before and after SHIV infection: SHIV-infected animals (*n* = 8) and SHIV-naïve animals (*n* = 7). **(G)** Frequency of rectal MAIT cells is maintained post-chronic SHIV infection (*n* = 22) compared to SHIV-naïve animals (*n* = 21). **(H)** Frequency of Ki-67+ MAIT cells in rectum before and after SHIV infection: SHIV-infected animals (*n* = 11) and (*n* = 7) SHIV-naïve animals. **(I)** Frequency of perforin+, granzyme-B+, and Ki67+perforin+granzyme-B+ MAIT cells in rectum are shown before and after SHIV infection: SHIV-infected animals (*n* = 11) and SHIV-naïve animals (*n* = 7). **(J)** Frequency of CD69+ MAIT cells in rectum before and after SHIV infection: SHIV-infected animals (*n* = 6) and SHIV-naïve animals (*n* = 7).

Previous studies have shown that there is a loss of peripheral MAIT cells during chronic HIV and SIV infection (12, 23); however, very few data demonstrated the functionality of these cells during chronic SIV infection in RM. Hence, we assessed MAIT cell frequency in the blood during chronic SHIV infection. Unlike what has been shown in chronic HIV/SIV infection, the frequency of blood MAIT cells is significantly higher (*p* = 0.02) than uninfected RM (Figure 3A). In line with the increased frequency of MAIT cells in the blood, these cells displayed higher proliferating capacity as defined by Ki-67 expression (Figure 3B). It is also important to note that MAIT cells in healthy humans are uniquely characterized by a lack of cytotoxic marker granzyme-B (12, 20). Since blood MAIT cells in SHIV-infected RM display high Ki-67, we also looked for their function by the expression of cytolytic markers perforin and granzyme-B. Interestingly, the MAIT cells maintain higher perforin levels, but the granzyme-B expression was not altered during SHIV infection. However, the total cytotoxic double-positive (perforin^+^Granzyme-B^+^) MAIT cells seems to be higher in the blood of SHIV-infected RM compared to the uninfected RM (Figure 3C). IL-18R expression marks functional MAIT cells (4, 33, 34); so we compared the expression of IL-18R expression on MAIT cells of SHIV-naïve and SHIV-infected RM. SHIV-infected RM displayed higher levels of IL-18R expression (Figure 3D). In order to understand the cytokine production capacity of MAIT cells during chronic SHIV infection, we stimulated total PBMC with PMA/ionomycin and looked for cytokine (IFN-γ, TNF-α, and IL-17) production. Cytokine production revealed that MAIT cells from the blood lose their capacity to make TNF-α and IL-17, whereas the IFN-γ producing capacity was maintained, suggesting that there is a restoration of IFN-γ-producing MAIT cells in the blood (Figure 3E). Recently, it has been shown that MAIT cells upregulate α4β7 (the gut homing integrin) during SIV infection in pigtail macaques (24); hence, we looked at the expression of α4β7 on RM blood MAIT cells during SHIV infection. Similar to the data on pigtail macaques, the expression of gut homing marker α4β7 was significantly higher on RM MAIT cells compared to non-MAIT cells post-SHIV infection (Figure 3F). Since MAIT cells from blood post-SHIV express α4β7, we looked at the rectal MAIT cell frequency post-SHIV infection. Although there is expression of α4β7, the frequency of rectal MAIT cells are not altered compared to uninfected RM (Figure 3G). Next we analyzed rectal MAIT cells for their proliferative and cytolytic potential. Unlike the blood MAIT cells, the rectal MAIT cells are not different for Ki67 (Figure 3H), perforin, or granzyme-B expression (Figure 3I). However, the total proliferating cytotoxic (Ki-67^+^perforin^+^granzyme-B^+^) MAIT cells are higher in rectal tissue in SHIV-infected RM compared to the uninfected RM. Similarly, the activation (CD69 expression) of rectal MAIT cells was higher compared to that of SHIV-naïve RM (Figure 3J). Collectively, these data suggest that, unlike the MAIT cells in uninfected RM that are mostly in resting stage (with low proliferation of cytotoxic potential such as Ki-67, granzyme-B, perforin, and CD69) (4), the MAIT cells present during chronic SHIV infection are highly activated, with phenotype showing high proliferative, cytotoxic, and IFN-γ producing capacity, and are functional.

### MAIT Cells Are Abundant in Non-lymphoid Tissues During Chronic SHIV Infection

**Figure 4:**
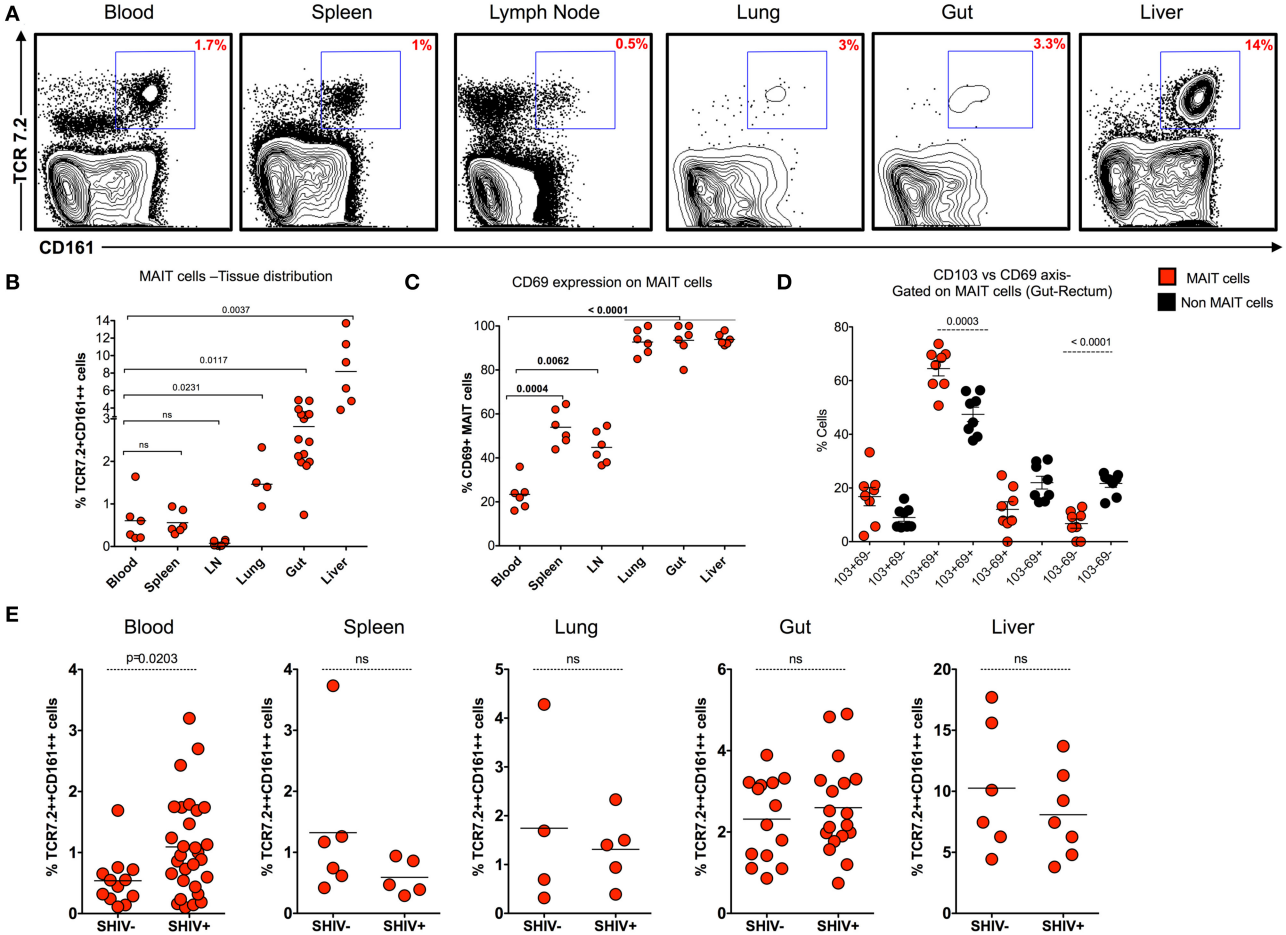
MAIT cells are abundant in non-lymphoid tissues during chronic SHIV infection. **(A)** Representative flow plots showing the expression of MAIT cells across different tissues post-SHIV infection. **(B)** Data showing the cumulative frequency of MAIT across all tissues. The liver shows the highest frequency of MAIT cells than any other tissue compartment. **(C)** Frequency of CD69+ MAIT cells post-SHIV infection. Non-lymphoid tissues express higher levels of CD69 marker than blood and secondary lymphoid tissues. **(D)** Frequency of rectal MAIT cells in CD103 and CD69 axis; data shown for SHIV-infected animals (*n* = 8). MAIT cells co-express higher levels of CD103 and CD69 than non-MAIT cells. **(E)** Data showing the frequency of MAIT cells between SHIV-naïve and chronically SHIV-infected animals in different compartments (blood, spleen, lung, rectum, and liver). No difference in tissue MAIT cells before and after SHIV infection.

MAIT cells are shown to be more abundant in tissues than in blood. In order to understand the distribution of MAIT cell in the tissues during chronic SHIV infection, we looked at the frequency of MAIT cells in different tissue compartments (blood, spleen, lymph node, lung, rectum, and liver) from chronic SHIV-infected RM (Figure 4A). Interestingly, we found that liver and rectum are enriched with a higher frequency of MAIT cells followed by lung, spleen, blood, and LN. Unlike the liver and rectum, MAIT cell frequencies were lower in the secondary lymphoid tissues (lymph node and spleen) (Figure 4B), suggesting that they are enriched preferentially in the non-lymphoid tissues. This is also evident based on the surface chemokine receptor expression of gut homing markers such as CCR6 and α4β7 in the blood (Figures 1F, 3F). We looked to see whether these cells are activated in the tissues by CD69 expression. Interestingly, MAIT cells from rectum, liver, and lung express significantly higher levels of CD69, unlike LN, spleen, and blood which scored less (<50%) for CD69 expression (Figure 4C). The CD69 expression on tissues strongly marks tissue-resident T cells despite their activation. Hence, in order to understand whether these cells are tissue resident, we looked for the co-expression profile of CD69 and CD103 (another marker for tissue residence). We determined the expression pattern in MAIT cells from rectum in a group of SHIV+ animals. We found that most of the CD69+ MAIT cells are CD103+, suggesting that these may be of tissue resident characteristic/phenotype (Figure 4D). A representative staining pattern for CD69 and CD103 co-expression is shown in Supplementary Figure 2A. It is also interesting to note that activated MAIT cells are present mostly in non-lymphoid tissues, which is one of the characteristics of tissue-resident memory T cells (which often express CD69 and CD103 and reside in non-lymphoid tissues) (35–37). We then compared the distribution of MAIT cells between SHIV-infected and uninfected RM in tissues. Notably, MAIT cell frequencies are not altered during chronic SHIV infection in tissues such as spleen, lung, rectum, and liver (Figure 4E). Altogether these data suggest that MAIT cells are abundant in tissues, in particular the liver and rectum, during chronic SHIV infection, which express activation/tissue-resident phenotypic makers and are mostly found in non-lymphoid tissues during chronic SHIV infection.

### MAIT Cells Correlate Inversely With SHIV Plasma Viral RNA Levels and Directly With CD4 T Cell Levels

**Figure 5:**
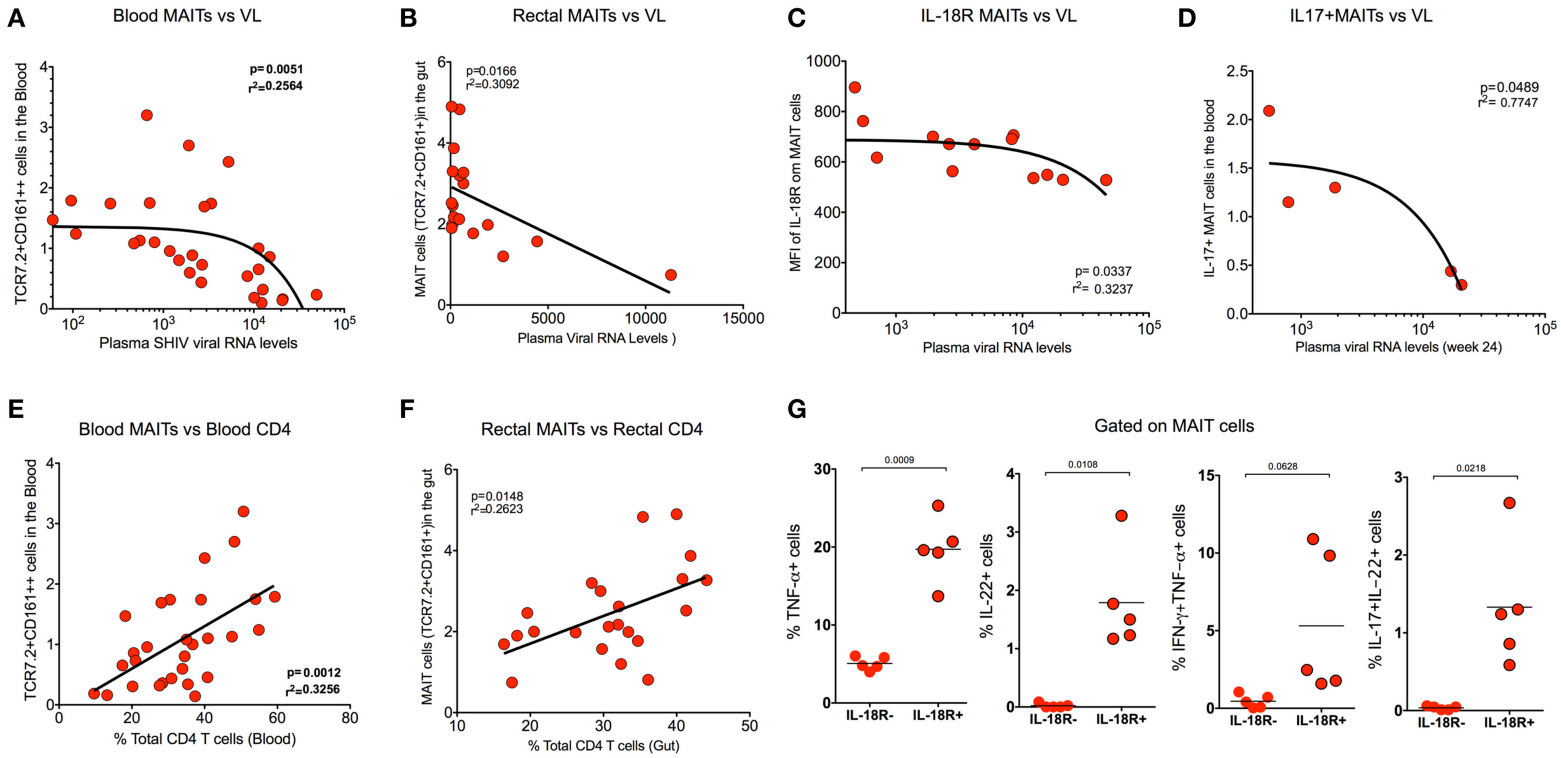
MAIT cells correlate inversely with SHIV plasma viral RNA levels and directly with CD4 T cell levels. **(A)** Plots showing the frequency of blood MAIT cells that correlated inversely with plasma SHIV viral RNA levels (*n* = 29). **(B)** Plots showing the frequency of gut MAIT cells that correlates inversely with plasma viral RNA levels (*n* = 18). **(C)** Plots showing the mean fluorescence intensity of IL-18R on blood MAIT cells that correlated inversely with plasma viral RNA levels (*n* = 13). **(D)** Plots showing the frequency of IL-17+ MAIT cells that correlates inversely with the plasma viral RNA levels (*n* = 5). **(E)** Plots showing the frequency of blood MAIT cells that correlates directly with the level of CD4 T cells (*n* = 29). **(F)** Plots showing the frequency of rectal MAIT cells that correlates directly with the level of CD4 T cells in the rectum (*n* = 24). **(G)** Plots showing the cytokines (TNF-α, IL-22, IFN-γ+TNF-α+, and IL-17+IL-22+ cells) produced by IL-18R+ and IL-18R- MAIT (IL-18R+CCR6+CD161++) cells after PMA/ionomycin stimulation (*n* = 5), suggesting that IL-18R expression is important for MAIT cell function during chronic SHIV infection.

In order to understand the association of MAIT cells with SHIV viral RNA levels, we compared the frequency of MAIT cells from blood with plasma viral RNA levels during chronic SHIV infection. Interestingly, the MAIT cell frequency displayed inverse correlation (*p* = 0.005) with plasma viral RNA levels in blood (Figure 5A). We compared the rectal MAIT cells and found that, similar to the blood, rectal MAIT cells also correlated inversely with SHIV plasma viral RNA levels (Figure 5B). In addition, post-SHIV infection blood MAIT cells express higher levels of IL-18R, which also correlated inversely with plasma SHIV RNA levels (Figure 5C). Studies have suggested that cytokines play a major role in regulating MAIT cell function (4, 7, 38). Since we observed that IL-18R expression correlated inversely with viral RNA levels, we wondered whether there is a significance of IL-18R-expressing MAIT cells. So, we compared the cytokine production capacity of IL-18R+ MAIT vs. IL-18R- MAIT cells. Interestingly, IL-18R+ MAIT cells produced significantly higher levels of cytokines than IL-18R- MAIT cells. However, significance was observed only in TNF-α, IL-22, and IL-17+IL-22+ cells, but not with IFN-γ and IL-17 (Figure 5G and Supplementary Figures 2D,E). Then, we looked at the association of cytokine+ MAIT cells vs. SHIV viral RNA levels. We did see an inverse correlation with the IL-17+ MAIT cells and plasma viral RNA levels (Figure 5D), but not with other cytokines such as IFN-γ and TNF-α (data not shown). CD4 T cells are important for CD8 T cell function (39). In order to understand the association of total CD4 T cell levels with MAIT cell frequency, we performed a correlation analysis of MAIT cells and total CD4 T cells from blood and rectal tissue during chronic SHIV infection. Interestingly, we observed a positive correlation of MAIT cells with CD4 T cell levels in both the blood and rectum of SHIV-infected RM (Figures 5E,F). Similar to the total CD4 T cells, we also see a direct correlation between Th17 (CCR6+CD4 T cells) from blood (Supplementary Figure 2B). Altogether these data clearly suggest that MAIT cells from SHIV-infected animals are functional and display significant inverse association with plasma RNA levels. We also believe that restoration of CD4 T cells during chronic HIV/SIV infection may restore functional MAIT cells during chronic SHIV infection.

### MAIT Cells Respond to Microbial Products During Chronic SHIV Infection

**Figure 6:**
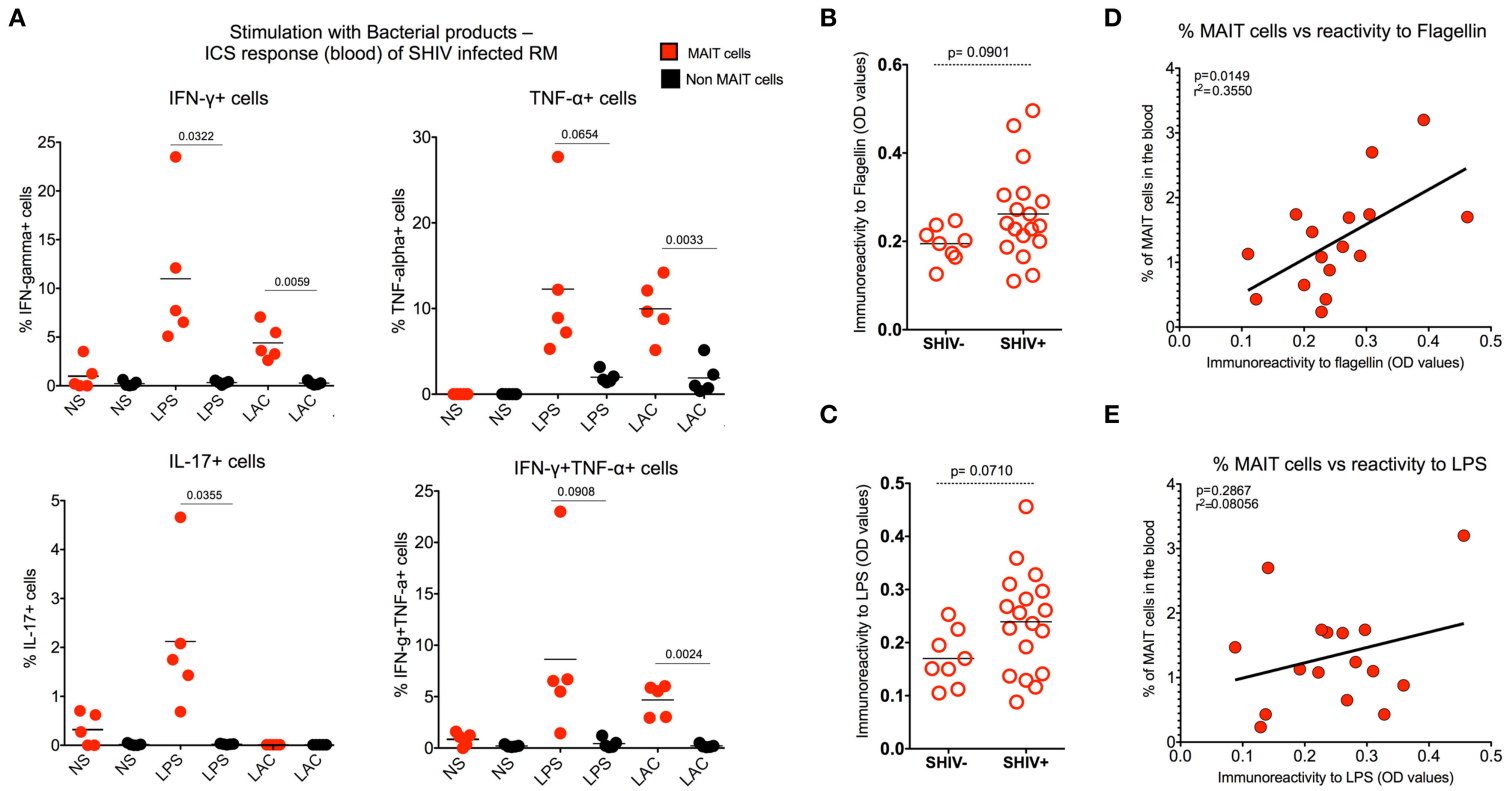
MAIT cells respond to microbial products during chronic SHIV infection. **(A)** Plots showing the cytokines (IFN-γ, TNF-α, and IL-17) produced by bacterial products (LPS and lactobacillus) stimulated cells (*n* = 5), suggesting antimicrobial immunity during chronic SHIV infection. **(B)** Dot plots showing the higher trend of reactivity to flagellin post-SHIV infection. **(C)** Dot plots showing the higher trend of reactivity to LPS post-SHIV infection (*n* = 17). **(D)** Plots showing that the frequency of blood MAIT cells correlates directly with the level of immunoreactivity to flagellin (*n* = 17). **(E)** Plots showing that the frequency of blood MAIT cells correlates directly with the level of immunoreactivity to LPS (*n* = 17).

It is also known that MAIT cells recognize a variety of microbial flora (1, 25), and considering the abundance of MAIT cells and their high activation at the mucosal surfaces and in the liver, the wide range of microorganisms that are able to produce the ligand for MR-1 may lead to MAIT cell activation (8, 40, 41). Hence, we determined whether MAIT cells are functional *in vivo* post-SHIV infection. MAIT cells were stimulated with bacterial products such as LPS or lactobacillus culture supernatants *in vitro* and tested for cytokine production during chronic SHIV infection as shown before (4, 32). We stimulated total PBMC of SHIV-infected RM overnight and measured the cytokine capacity of MAIT cells using ICCS assay (11, 42). We observed that bacterial products enhanced the cytokine (IFN-γ, TNF-α, and IL-17) producing capacity of MAIT cells but not non-MAIT cells (Figure 6A). Given that microbial translocation may stimulate innate immune cells via TLR pathways (10, 27), leading to immune activation, and given the well-recognized role of microbial translocation during chronic HIV and SIV infection (43–48), we explored the possible association between MAIT cells and the markers of microbial translocation (such as flagellin and LPS) during chronic SHIV infection. We found a trend toward increasing pattern (yet not significant) of serum levels of immunoreactivity to flagellin and LPS between SHIV-naïve and SHIV-infected macaques (Figures 6B,C). Although the trend is not significant, the MAIT cell frequency in the blood correlated directly with flagellin and a trend toward immunoreactivity to LPS levels (Figures 6D,E). These data could argue that bacterial products may have a role in activating MAIT cells *in vivo* during chronic SHIV infection. Based on these data, we believe that the enhancement of MAIT cell frequency in the blood may be due to the accumulation of bacterial products post-SHIV infection *in vivo*.

### Cytokine IL-7 Maintains the Proliferation but IL-12 and IL-18 Maintains the Cytotoxic Capacity of MAIT Cells During Chronic SHIV Infection

**Figure 7:**
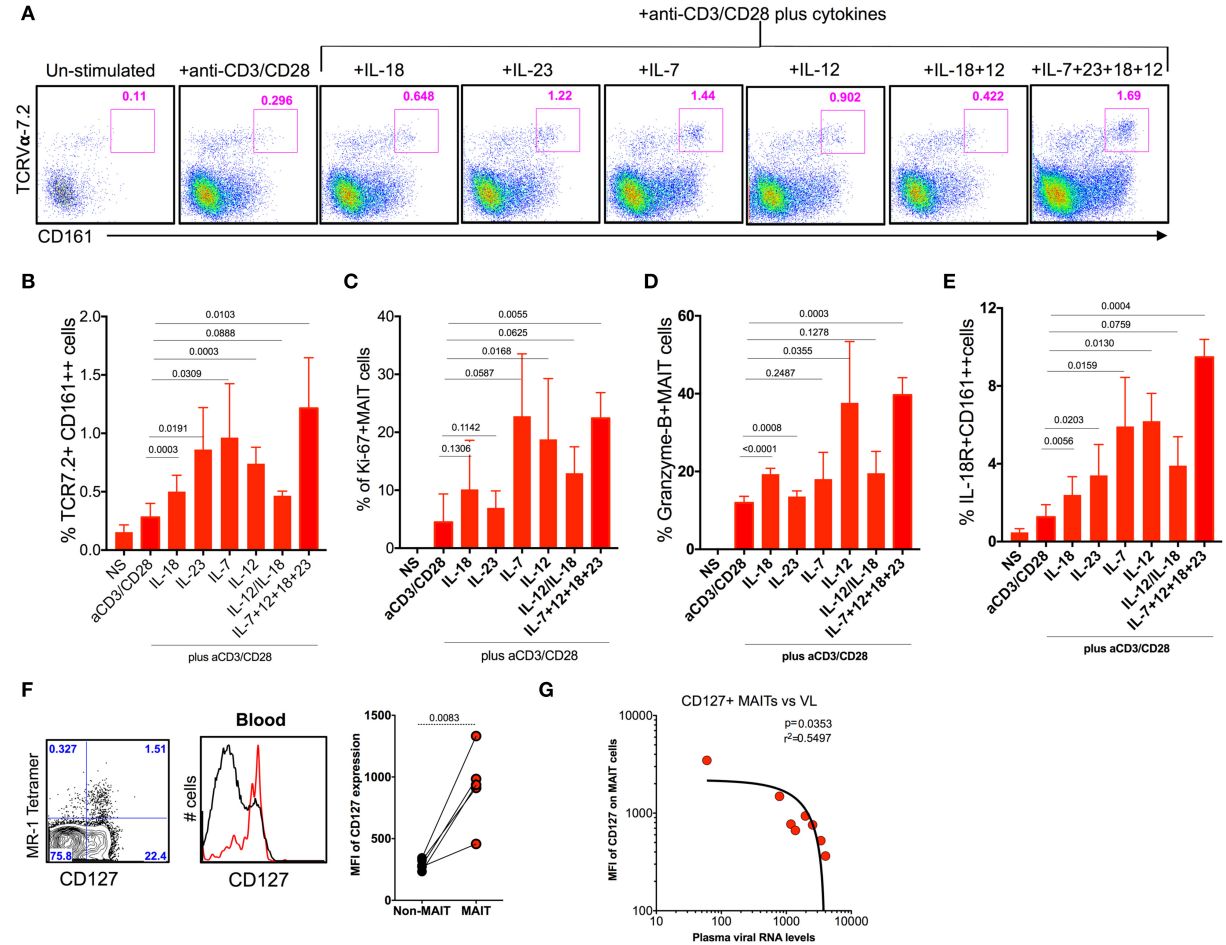
Cytokine IL-7 maintains the proliferation, and IL-12 and IL-18 maintain the cytotoxic capacity of MAIT cells. **(A)** Representative flow plots showing the induction of MAIT cell markers after anti-CD3/anti-CD28 stimulation with and without the addition of cytokines. **(B)** Plots showing the cumulative data for the frequency of MAIT cells after anti-CD3/anti-CD28 stimulation with and without the addition of different cytokines. **(C)** Plots showing the frequency of proliferating MAIT cells after anti-CD3/anti-CD28 stimulation with and without the addition of different cytokines. **(D)** Plots showing the frequency of granzyme-B+ MAIT cells after anti-CD3/anti-CD28 stimulation with and without the addition of different cytokines. **(E)** Plots showing the frequency of IL-18R expressing MAIT cells after anti-CD3/anti-CD28 stimulation with and without the addition of different cytokines. **(F)** Representative flow plots showing the expression of IL-7R on MAIT cells. **(G)** IL-7R expressing MAIT cells correlates inversely with plasma viral RNA levels (*n* = 8).

MAIT cells express various cytokine receptors such as IL-18, IL-12, IL-7, and IL-23 (9), which may be modulated during chronic HIV/SIV infection. In order to determine what cytokines are important for MAIT cell activation/function during chronic SHIV infection, we stimulated total PBMC from SHIV-infected RM with different cytokines (IL-7, IL-12, IL-23, and IL-18) either alone or as a combination of all cytokines with and without anti-CD3/CD28 stimulation for 5 days and looked at the frequency, activation (IL-18R), proliferation (Ki-67), and cytolytic capacity (granzyme-B) of MAIT cells from (*n* = 4) SHIV-infected RM. Interestingly, among the cytokines tested, IL-7 seemed to stimulate MAIT cells better compared to others; however, IL-23, IL-12, and a combination of all cytokines enhanced the frequency of MAIT cells significantly (Figures 7A,B,E), suggesting that treatment with these cytokines enhances the frequency of MAIT cells *in vivo*. In order to understand the effect of cytokines on the proliferation and cytolytic capacity of MAIT cells, we looked at the expression of Ki-67 and granzyme-B on these expanded MAIT cells (Figures 7C,D). Interestingly, cytokines IL-7 and IL-12 induce the proliferation of (Ki-67) MAIT cells, or the combination of all cytokines (IL-18, IL-7, IL-23, and IL-12) enhanced the proliferation capacity of MAIT cells. There is no significant difference for proliferation with cytokine IL-23 or IL-18. Unlike proliferation, the cytotoxic capacity of MAIT cells was enhanced with cytokines IL-12 and IL-18 but not with IL-7 or IL-23, suggesting that each cytokine stimulates different functional properties of MAIT cells. We also looked at the IL-18R expression on the activated MAIT cells. It indicates that IL-7 and IL-12 induced the expression of IL-18R to high levels (Figure 7E). More interestingly, the combination of cytokines IL-7, IL-12, IL-23, and IL-18 did increase the frequency, proliferation, and cytolytic capacity of MAIT cells during chronic SHIV infection, suggesting that this combination of cytokines as treatment for HIV/SIV during chronic infection may induce functional MAIT cells *in vivo*. It is important to note that similar effects were seen with cytokines in SHIV-naïve animals (Supplementary Figures 1E–G). Since IL-7 is important for MAIT cell proliferation, we also looked at the expression of IL-7R on MAIT cells during chronic SHIV infection. MAIT cells express very high levels of IL-7R (Figure 7F), and IL-17R expression inversely correlated with plasma viral RNA levels (Figure 7G). These data are in line with published HIV data (49) that MAIT cells express higher levels of IL-7Ra and IL-18R. Overall these data collectively suggest that cytokines may modulate MAIT cell function *in vivo* and further indicate that cytokine-specific induction of functional MAIT cells can occur *in vivo* during chronic SHIV infection.

## Discussion

In the current study, we used a RM model of chronic SHIV infection and evaluated the frequency, phenotype, distribution, and function of MAIT cells in blood and rectum and its association with viral RNA levels. Here we used both rhesus MR1 tetramer and human MAIT cell markers (CD3^+^ CD8^+^CD161^++^Vα7.2^+^) as a marker for MAIT cell identification in RM. The combination of markers CD8^+^CD161^++^Vα7.2^+^ identified the majority of MR1 tetramer-positive MAIT cells (2). The MR1 staining pattern correlates very strongly with the frequencies from the CD161^++^Vα7.2^+^ staining pattern in CD3+ CD8+ T cells. This offers a comprehensive phenotypic analysis of RM MAIT cells and displays a phenotypic similarity between human and macaque MAIT cells. Macaque MAIT cells express markers similar to those of human MAIT cell such as IL-18R^hi^, CCR6^hi^, CD69^hi^, CD28^hi^, and CD95^hi^ and express high levels of transcription factor PLZF^hi^ (6, 8, 9, 50). Notably, these cells are mostly of memory phenotype in RM unlike the non-MAIT cells, suggesting that these cells are ready to expand post-antigen stimulation *in vivo*.

Studies in chronic HIV infection have documented that MAIT cells are depleted in peripheral blood (9, 22, 51). Similar to HIV infection, it has been shown that MAIT cells are depleted systemically during chronic SIV infection in RM (23). In contrast with the HIV and SIV RM studies, recent data from pigtail macaques demonstrated an accumulation of α4β7+ MAIT cells in the rectal mucosa during chronic SIV infection (24). Our results suggest that blood MAIT cells from SHIV-infected RM are higher compared to SHIV-naïve RM. These MAIT cells in SHIV-infected macaques display an activated phenotype during chronic SHIV infection. The activated phenotype of MAIT cells during SHIV infection resembles the phenotype observed during certain viral infections (influenza, DENV, and HCV) (52, 53) and bacterial infections (26, 40, 54), which express higher levels of IL-18R, CD69, IFN-gamma, granzyme-B, perforin, and Ki-67 (10, 53). Chronic HIV-infected and HCV-infected individuals display reduced CD161+ cells on MAIT cells (55, 56). However, during chronic SHIV infection, we did not see the downregulation of CD161+ MAIT cells post-SHIV infection (Supplementary Figure 2C). Since we know that CD161 marks functional CD8 T cells (57, 58), we believe that intact CD161 expression may render the functional quality of MAIT cells *in vivo* (4). Similar to the data published in pigtail macaques during SIV/SHIV infection, we also see a higher expression of α4β7 on MAIT cells in the blood post-SHIV infection; however, the MAIT cell frequency is comparable in the rectum between uninfected and SHIV-infected RM. The mechanism that drives the increase in blood MAITs and unaltered rectal MAITs after α4β7 expression on blood MAITs is not clear; however, we think that it may be a result from translocation of microbial products from the gut to the peripheral compartment or may be a result of intact CD4 T cells during chronic SHIV infection in the blood and rectum. These data are consistent with the data obtained from SIV-infected pigtail macaques (24) (they show an increase in α4β7+MAIT cells in the rectum), but this is in contrast with SIV-infected RM (23). Although MAIT cell frequency is similar in the rectum, these cells are activated in the rectal mucosa and are polyfunctional with their ability to make perforin and granzyme-B with high proliferative capacity during chronic SHIV infection. Having shown activated MAIT cell phenotypes during chronic SHIV infection, we next asked whether their anti-bacterial properties are intact by analyzing the immunoreactivity to serum LPS and flagellin levels as well as stimulating MAIT cells with bacterial products. Interestingly, MAIT cells reacted to microbial products during chronic SHIV infection. In addition, there is no difference between SHIV-infected and uninfected macaques with the *ex vivo* reactivity of MAIT cells cultured together with LPS, implying intact antibacterial functions of MAITs during chronic SHIV infection. Indeed the association of MAIT cells with bacterial product flagellin as well as the cytokine production with LPS stimulation suggests that it may have a role in triggering MAIT cells *in vivo* during chronic SHIV infection. We strongly feel that low levels of activation with microbial products might play as an activator of MAIT cells during chronic SHIV infection. However, the mechanism behind this should be studied further. It is also interesting to note that MAIT cells express activation/tissue-resident marker CD69 at higher levels in non-lymphoid tissues (lung, gut, and liver) than lymphoid tissues (spleen and LN) during chronic SHIV infection. This may also suggest that these MAIT cells are retained in the tissues that express high levels of activation/retention marker CD69 and CD103. In addition, it is also clear that these cells are not depleted in the tissues during chronic SHIV infection, which is in contrast to what have been seen with SIV infection (23). The mechanism behind the expansion of MAIT cells in blood and maintenance of these cells in tissues during chronic SHIV infection is not known clearly and needs to be studied further.

Cytokines activate MAIT cells independently of their TCR (4), and MAIT cells are shown to express high levels of interleukin receptors (IL-18R, IL-12R, IL-7R, and IL-23R) (9). Treatment with cytokines during chronic SHIV infection showed that cytokine IL-7 might play a significant role in inducing MAIT cell frequencies in high levels. This is consistent with human data, which demonstrated that administration of IL-7 *in vivo* enhances the frequency and quality of MAIT cells during chronic HIV infection (49). These studies suggest that the IL-7 pathway could be modulated *in vivo* to augment the function of MAIT cells during chronic HIV infection. One important point we would like to note is that *in vitro* TCR stimulation has been shown to reduce the expression of CD161 on TCR7.2+ T cells (59). However, we did not see a similar trend in our *in vitro* anti-CD3/CD28 TCR stimulation experiments in SHIV-infected macaques. Although this is intriguing, it is important to know whether this phenomenon is true with other proliferation experiments such as the CFSE/other dye assays to assess MAIT cell proliferation directly since TCR stimulation also induce the activation/proliferation of many other T cell subsets. In addition to proliferation, we also see a difference in the functional capacity of MAIT cells between cytokines. Although IL-7 expanded the MAIT cell frequencies, the cytolytic capacity of MAIT cells was enhanced with the cytokines IL-12 and IL-18 but not with IL-7, suggesting that each cytokine stimulates different functional properties of MAIT cells. The correlation between levels of IL-18R and IL-7R expression and MAIT cell frequency suggests an important role for these cytokines (IL-18 and IL-7) in the induction/maintenance of MAIT cell function *in vivo* during chronic SHIV infection. Interestingly, the synergistic effects of all cytokines including IL-7, IL-12, IL-23, and IL-18 did increase the total frequency, proliferation, and cytolytic capacity of MAIT cells, suggesting that a combination of these cytokines as treatment during chronic HIV infection may induce functional MAIT cells *in vivo*.

Overall our data suggest that MAIT cells in the rectum are not reduced during chronic SHIV infection, but they maintained their functionality. We believe that this polyfunctionality was due to the help from CD4 T cells *in vivo* since MAIT cell frequency correlated directly with the CD4 T cell levels during chronic SHIV infection both in blood and rectum, which is not a characteristic in SIV-infected RM or in HIV-infected humans. The most interesting data is the association of MAIT cell frequencies with SHIV viral load. Animals with lower viral levels exhibited higher functional markers such as IL-7R and IL-18R, and animals with higher IL-17 production had lower viral levels. We believe that IL-7R- and IL-18R-expressing MAIT cells may be functional *in vivo*. These data also suggest that immunoregulatory mechanisms that occur during chronic HIV infection may potentially influence the phenotypic and functional properties of MAIT cells *in vivo* since we see an expansion of functional MAIT cells with these (IL-7 and IL-18) cytokines *in vitro*. In addition, the blood MAIT cells during chronic SHIV infection responded with bacterial products such as LPS (TLR4 agonist) and lactobacillus, suggesting that MAIT cells during chronic SHIV infection have the functional quality to respond to bacterial products *in vivo*, which in turn enhances gut integrity that may have contributed to viral reduction. Further investigation into early immune signatures is needed to help in addressing what mechanisms influence the *in vivo* generation of MAIT cells and their function. Therapeutic strategies that maintain or elicit the levels of these cytokines *in vivo* during chronic HIV/ SIV infection may benefit MAIT cells in order to regulate gut homeostasis during chronic HIV/SIV infection. In addition, it is very important to understand the mechanisms underlying the induction of MAIT cells by vaccination and during infection in order to develop immune-based strategies that can substantially reduce the immune activation and gut pathology early post-HIV infection. Overall our data suggest that MAIT cells are activated during chronic SHIV infection and are negatively associated with SHIV viral RNA levels. Therapeutic strategies such as cytokine therapies may be beneficial in inducing functional MAIT cells for reducing SIV/HIV infection as well as other opportunistic co-infections *in vivo*.

## Materials and Methods

### Ethics Statement

All animal experimentations were conducted at the Yerkes National Primate Research Center, which is accredited by the American Association of Accreditation of Laboratory Animal Care International, following guidelines established by the Animal Welfare Act and the NIH for housing and care of laboratory animals. Blood and tissue collections were obtained under anesthesia. RM were fed standard monkey chow (Jumbo Monkey Diet 5037, Purina Mills, St. Louis, MO) supplemented with fresh fruit or vegetable daily. Consumption is monitored and adjustments are made as necessary depending on sex, age, and weight. SIV-infected RMs were singly caged but had visual, auditory, and olfactory contact with at least one social partner, permitting the expression of non-contact social behavior. The YNPRC enrichment plan employs several general categories of enrichment. This study was performed in strict accordance with the recommendations in the Guide for the Care and Use of Laboratory Animals of the National Institutes of Health, a national set of guidelines in the US, and also to international recommendations detailed in the Weatherall Report (2006). This work received prior approval by the Institutional Animal Care and Use Committee (IACUC) of Emory University. Appropriate procedures were performed to ensure that potential distress, pain, discomfort, and/or injury was limited to that unavoidable in the conduct of the research plan. Ketamine (10 mg/kg) and/or telazol (4 mg/kg) was used for collection of blood and tissues, and analgesics were used when determined appropriate by the veterinary medical staff.

### Animals

A total of 43 animals (12 SHIV-naive and 31 chronically SHIV 1157ipd3N4-infected Indian adult rhesus macaques) obtained from the Yerkes breeding colony were cared for under the guidelines established by the Animal Welfare Act and the National Institute of Health (NIH) Guide for the Care and Use of Laboratory Animals using protocols approved by the Emory University Institutional Animal Care and Use Committee. SHIV-infected animals were infected with SHIV1157ipd3N4 at a dose of 700 TCID_50_ intrarectally. Some animals were positive for Mamu A^*^01 allele, and all animals were negative for Mamu B08 and Mamu B17 (see Table 1).

**Table 1:** Cohort of SHIV infected rhesus macaques used for the study.

**Animal name**	**Set-point viral load (#)**	**Challenge virus**	**Route of infection**	**Mamu A01 status**	**Sex**	**Week of sampling**	**Animals used in figures**
32034	N/A	N/A	NA	N/A	Male	N/A	1
34909	N/A	N/A	NA	N/A	Male	N/A	1
34943	N/A	N/A	NA	N/A	Male	N/A	1
32113	N/A	N/A	NA	N/A	Male	N/A	1
33104	N/A	N/A	NA	N/A	Male	N/A	1
RQi5	N/A	N/A	NA	N/A	Female	N/A	1, 2
RJq16	N/A	N/A	NA	N/A	Female	N/A	1, 2
REo16	N/A	N/A	NA	N/A	Female	N/A	1, 2
RAm16	N/A	N/A	NA	N/A	Female	N/A	1, 2
RRq16	N/A	N/A	NA	N/A	Female	N/A	1, 2
RFi16	N/A	N/A	NA	N/A	Female	N/A	1, 2
RAp16	N/A	N/A	NA	N/A	Female	N/A	1, 2
224-12	60	SHIV1175	Intra-rectal	–	Female	24	3, 5, 6
RJb15	60	SHIV1175	Intra-rectal	–	Male	24	3, 5
RKe16	2540	SHIV1175	Intra-rectal	_**+**_	Female	24	3, 5, 6
29-12	3400	SHIV1175	Intra-rectal	–	Female	24	3, 5, 6
RTs15	1970	SHIV1175	Intra-rectal	–	Male	24	3, 5, 6
RKe15	1180	SHIV1175	Intra-rectal	–	Male	24	3, 5, 6
145-12	1380	SHIV1175	Intra-rectal	–	Male	24	3, 5, 6
RVj15	11300	SHIV1175	Intra-rectal	–	Male	14	3, 5, 6
RCj15	108	SHIV1175	Intra-rectal	_**+**_	Male	14	3, 5, 6
RAj15	659	SHIV1175	Intra-rectal	_**+**_	Male	14	3, 5, 6
RKe-16	15000	SHIV1175	Intra-rectal	–	Female	14	3, 4, 5
RJf16	5260	SHIV1175	Intra-rectal	_**+**_	Male	14	3, 5, 7
RPc15	1913	SHIV1175	Intra-rectal	–	Male	14	3, 5, 6
RUd15	60	SHIV1175	Intra-rectal	–	Male	14	3, 5, 6
RTf15	2680	SHIV1175	Intra-rectal	–	Male	14	3, 5, 7
29122	20900	SHIV1175	Intra-rectal	–	Female	14	3, 5
RKe16	1180	SHIV1175	Intra-rectal	–	Female	14	3, 5, 7
22412	12200	SHIV1175	Intra-rectal	–	Female	14	3, 5
146-12	3390	SHIV1175	Intra-rectal	_**+**_	Male	14	3, 5, 6
RDi15	96	SHIV1175	Intra-rectal	_**+**_	Male	14	3, 5, 6
RIf16	260	SHIV1175	Intra-rectal	–	Male	14	3, 5, 6
RVl15	550	SHIV1175	Intra-rectal	_**+**_	Male	14	3, 5, 6
RIb16	2090	SHIV1175	Intra-rectal	–	Male	14	3, 5, 6
RKi15	1960	SHIV1175	Intra-rectal	–	Male	14	3, 5, 6
RJy15	800	SHIV1175	Intra-rectal	–	Female	14	3, 5, 6
RJb11	12600	SHIV1175	Intra-rectal	–	Male	14	3, 5, 6, 7
RVj11	11420	SHIV1175	Intra-rectal	–	Male	14	3, 5, 6
RLr15	474	SHIV1175	Intra-rectal	–	Male	14	3, 5, 6
RTs12	1490	SHIV1175	Intra-rectal	–	Male	14	3, 5, 6
RKe12	2630	SHIV1175	Intra-rectal	–	Male	14	3, 5, 6
RJv15	708	SHIV1175	Intra-rectal	–	Male	14	3, 5, 6

*+ Meaning Mamu A01+ animals*.

### Antibodies

Cells were stained with fluorochrome-conjugated Abs specific for CD3 (clone SP-34-2; BD Biosciences), CCR6 (clone 11A9), CD95 (clone DX2; BD Biosciences), PD-1 (Clone EH12.1; Biolegend), CD8 (Clone SK1; BD Bioscience), CD4 (clone; OKT4; Biolegend), α4β7 (Act1, NHP reagent resource), LIVE/DEAD fixable Near-IR Dead Cell stain (Invitrogen), Ki-67 (Clone B56; BD Biosciences), IFN-γ (Clone B27; BD Biosciences), CD95 (Clone DX2; BD Biosciences), CD28 (Clone 28.2; BD Biosciences), IL-17A (eBio64DEC17; eBioscience), TNF-α (MAb11; BD biosciences), IL-22 (IL22JOP; ebiosciences), IL-7R (Clone eBioRDR5; eBioscience), CD161 (HP3G10; Biolegend), TCR7.2 (3C10; Biolegend), CD69 (FN50; Biolegend), IL-18Rα (H44; Biolegend), streptavidin-BV421- conjugated MR1 tetramer (NIH tetramer core facility), Granzyme B (Clone GB1; BD Biosciences 1), and perforin (Clones Pf-80/164; BD Biosciences).

### Phenotyping

Mononuclear cells were stained with LIVE/DEAD Near-IR Dead Cell at room temperature for 15 min in PBS to stain for dead cells. Cells were then washed with FACS wash, stained on the surface using antibodies specific to respective cell markers, then treated with 1× BD FACS lysing solution for 10 min at room temperature, permeabilized with 1× BD permeabilizing solution for 8 min at room temperature, washed with FACS wash, stained intracellularly using antibodies specific to the respective intracellular markers, washed twice with FACS wash, and assessed by flow cytometry as described previously (60, 61).

### Intracellular Cytokine Staining

Mononuclear cells were isolated from the blood, axillary lymph nodes, rectal tissue, bronchoalvelolar lavage, spleen, and rectum, and flow cytometry analysis was performed as described previously (36). Stimulations were conducted in the presence of anti-CD28 antibody and anti-CD49d antibody (1 μg/mL; BD Pharmingen). One million cells were stimulated with 80 ng/mL PMA and 1 μg/mL ionomycin as a positive control. Brefeldin A (5 μg/mL; Sigma) and GolgiStop (0.5 μL/mL; BD Pharmingen) were then added to the cells after 2 h of stimulation, and incubation was continued for 4 h at 37°C in the presence of 5% CO_2_. At the end of stimulation, cells were washed once with FACS wash [PBS containing 2% (vol/vol) FBS and 0.25% of sodium azide] and surface-stained with anti-CD8, anti-CD4, anti-CD161, anti-IL18Rα, TCR 7.2, and LIVE/DEAD Near-IR Dead Cell stain (Life Technologies) at room temperature for 20 min. Cells were then fixed with cytofix/cytoperm (BD Pharmingen) for 20 min at 4°C and washed with Perm wash (BD Pharmingen). Cells were then incubated for 30 min at 4°C with antibodies specific to IL-17, IFN-γ, TNF-α, IL-22, and CD3, washed once with Perm wash and once with FACS wash, and resuspended in PBS containing 1% formalin. Cells were acquired on LSR-Fortessa with four lasers (405, 488, 532, and 633 nm) and analyzed using the FlowJo software (Treestar Inc.). At least 50,000 events were acquired for each sample as described previously (60, 61).

### Bacterial Stimulation

Rhesus PBMCs (1 × 10^6^) were incubated with LPS (10 ng/ml) and with lactobacillus culture supernatant (10 μl) for a final 6 h of culture. Brefeldin A (10 μg/ml) was added for the final 4 h of culture, and cells were stored overnight in 4°C until stained. Cells were analyzed for cytokine production by ICS assay as described previously (62).

### Quantitation of SHIV RNA

The SHIV copy number in plasma was determined using quantitative real-time PCR as described previously (60).

### *In vitro* Proliferation Assay

PBMCs were cultured in 10 ng/ml recombinant human IL-7 (R&D Systems), IL-12 (10 ng/ml), IL-23 (5 ng/ml), IL-18(10 ng/ml), or a combination of all cytokines with LPS as positive control (100 ng/ml) for 5 days as indicated or left untreated at 37°C and 5% CO_2_ in RPMI medium supplemented with 10% fetal calf serum and 50 μg/ml gentamicin (Gibco) (RF10 medium). Following 6 days of culture, appropriate surface (CD3, CD8, CD4, TCR7.2, and CD161) staining and intracellular staining with Ki-67 and Granzyme-B were performed as described earlier (61).

### Serum Immunoreactivity to LPS and Flagellin

Serum immunoreactivity to LPS and flagellin was examined by ELISA as described previously by Ziegler et al. (63). High-binding ELISA plates were coated overnight at 4°C with purified flagellin (100 ng/well) or LPS (2 μg/well; from *Escherichia coli* 0128: B12, Sigma) in 9.6 pH bicarbonate buffer. Sera were diluted 1:250 or 1:500 in wash buffer (0.05% goat serum and 0.01% Tween 20 in PBS) and added to wells coated with flagellin or LPS after washing once with wash buffer. After incubation at 37°C for 1 h, the wells were washed thrice and then incubated with HRP-conjugated anti-monkey IgG (1:10,000). After washing three times, the peroxidase substrate tetramethylbenzidine was added to the wells, and after 5–20 min, optical density (OD) was read at 450 nm with an ELISA plate reader. Data are reported as OD corrected by subtracting with the readings in blank samples.

### Statistical Analyses

A paired *t*-test was used for comparisons between two or more subsets from the same animal. Unpaired *t*-test was used for comparisons between uninfected and SHIV-infected animals. Spearman rank test was used for all correlations. Boolean analyses were performed using SPICE software (NIAID, NIH). GraphPad Prism statistical analysis program (GraphPad Software) was used to determine the *P*-values. *P* < 0.05 were considered significant.

## Data Availability Statement

All datasets generated for this study are included in the article/Supplementary Material.

## Ethics Statement

The animal study was reviewed and approved by Emory IACUC.

## Author Contributions

VV and RA contributed to the concept and design of experiments. VV supervised the project and coordinated the experiments. VV, AM, and CI performed the experiments. VV, US, and CI analyzed the data. TS, PR, and AJ provided PBMC and rectal samples for MAIT cell analysis from SHIV-naïve animals. CI, SR, and US provided help with *in vitro* experiments with cytokines. PS and MV-K performed experiments on flagellin and LPS. VV wrote and edited the manuscript. All authors reviewed the manuscript and discussed the work.

### Conflict of Interest

The authors declare that the research was conducted in the absence of any commercial or financial relationships that could be construed as a potential conflict of interest.

## Supplementary Material

Supplementary Figure 1 **(A)** Representative Facs plots showing the staining pattern of tetramers 6-FP and 5-A-RU from two SHIV-naïve rhesus macaques. **(B)** Distribution of MR-1 tetramer within different T cell subsets; CD8 expressed >94% MR1. **(C)** Representative Facs plots showing the frequency of MAIT cells and relative levels of transcription factor PLZF in tissues between MAIT and non-MAIT cells. **(D)** Facs plot showing the MR-1 staining pattern in liver. **(E)** Representative flow plots showing the staining pattern/induction of MR-1+CD161+CD8, MR-1+TCR7.2+ CD8 T cells after anti-CD3/anti-CD28 stimulation with and without the addition of cytokines in SHIV-naïve animals. **(F)** Plots showing the cumulative data for the frequency of MAIT cells (MR-1+CD161+ cells) after anti-CD3/anti-CD28 stimulation with and without the addition of different cytokines (*n* = 2). **(G)** Plots showing the frequency of proliferating (Ki-67+) MAIT cells (MR-1+CD161+ cells) after anti-CD3/anti-CD28 stimulation with and without the addition of different cytokines (*n* = 2). IL-7 seems to enhance the proliferation of MAIT cells in SHIV-naïve animals.

Supplementary Figure 2 **(A)** Representative Facs plots showing the staining pattern of tissue-resident markers CD69 and CD103 on rectal MAIT and non-MAIT cells from a SHIV-infected RM. **(B)** Plots showing the positive correlation between the Th17 cells (CCR6+CD4+ T cells) vs. MAIT cells in SHIV-infected macaques. **(C)** Representative Facs plots showing the staining pattern on MR-1 vs. CD161 from five SHIV-infected macaques. **(D)** Representative Facs plots showing the production of cytokines (IFN-γ, TNF-α, IL-17, IL-22, IFN-γ+TNF-α+, and IL-17+IL-22+ cells) by IL-18R+ and IL-18R-ve MAIT cells during chronic SHIV infection in an animal. **(E)** IL-18R expression did not show any difference in IFN-γ+ or IL-17+ single positive cytokine (*n* = 5).
