# Supplementary figures and images for: Functional MAIT Cells Are Associated With Reduced Simian–Human Immunodeficiency Virus Infection
Source: Front Immunol. 2020 Jan 17;10:3053. doi: 10.3389/fimmu.2019.03053 (PMC6978843; doi:10.3389/fimmu.2019.03053)

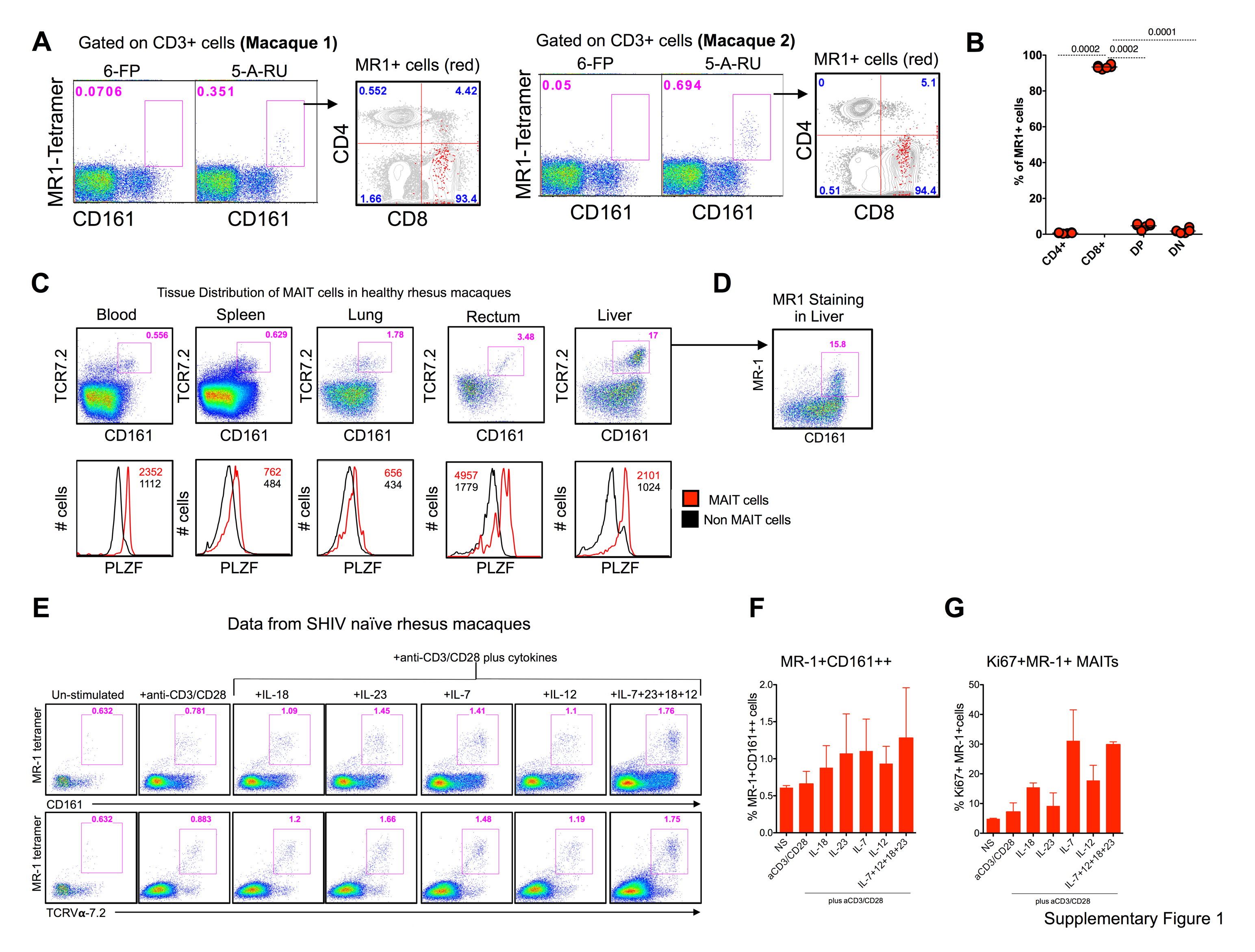

Supplement: Supplementary Figure 1 — (A) Representative Facs plots showing the staining pattern of tetramers 6-FP and 5-A-RU from two SHIV-naïve rhesus macaques. (B) Distribution of MR-1 tetramer within different T cell subsets; CD8 expressed >94% MR1. (C) Representative Facs plots showing the frequency of MAIT cells and relative levels of transcription factor PLZF in tissues between MAIT and non-MAIT cells. (D) Facs plot showing the MR-1 staining pattern in liver. (E) Representative flow plots showing the staining pattern/induction of MR-1+CD161+CD8, MR-1+TCR7.2+ CD8 T cells after anti-CD3/anti-CD28 stimulation with and without the addition of cytokines in SHIV-naïve animals. (F) Plots showing the cumulative data for the frequency of MAIT cells (MR-1+CD161+ cells) after anti-CD3/anti-CD28 stimulation with and without the addition of different cytokines (n = 2). (G) Plots showing the frequency of proliferating (Ki-67+) MAIT cells (MR-1+CD161+ cells) after anti-CD3/anti-CD28 stimulation with and without the addition of different cytokines (n = 2). IL-7 seems to enhance the proliferation of MAIT cells in SHIV-naïve animals. [file Image_1.TIFF]

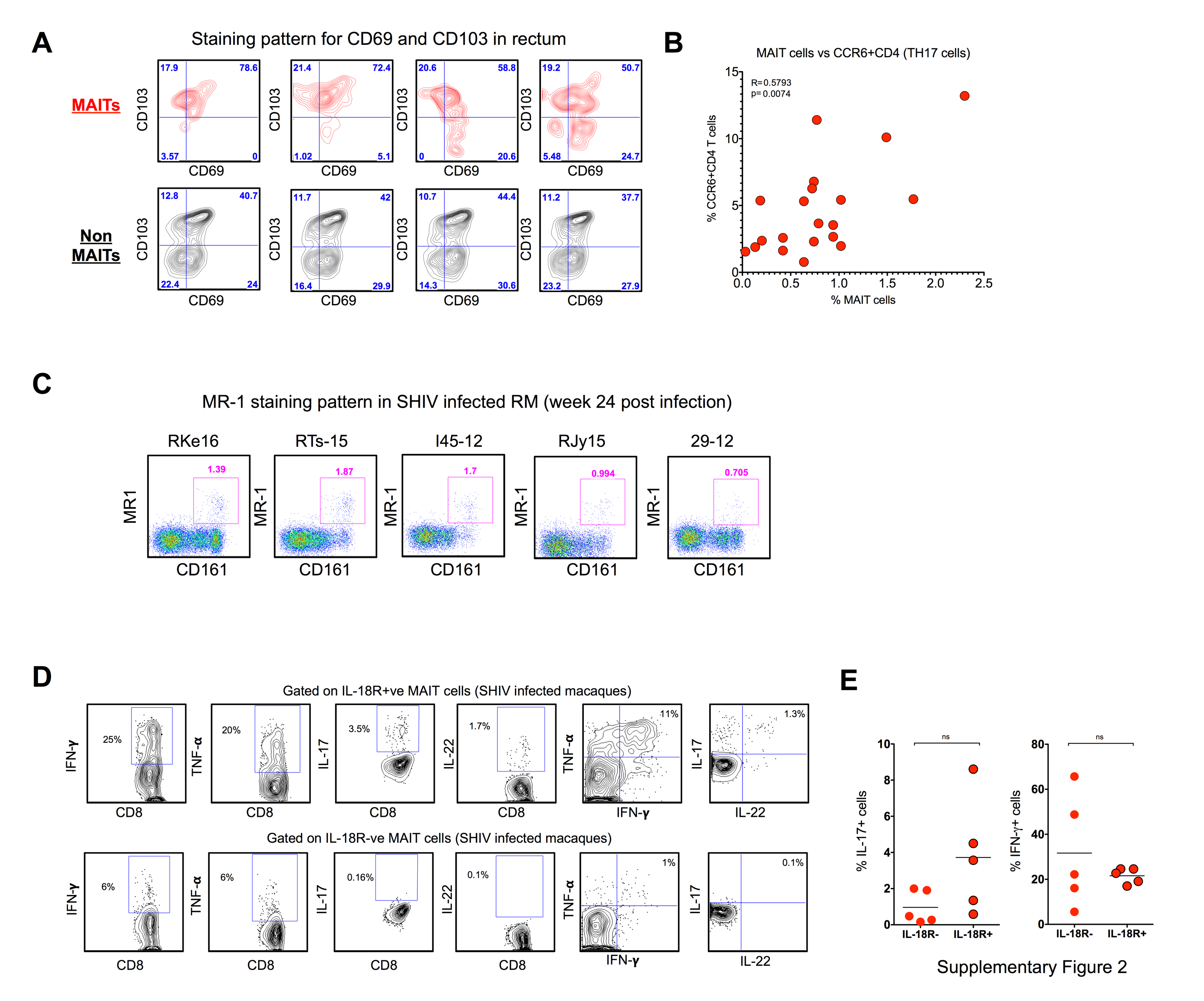

Supplement: Supplementary Figure 2 — (A) Representative Facs plots showing the staining pattern of tissue-resident markers CD69 and CD103 on rectal MAIT and non-MAIT cells from a SHIV-infected RM. (B) Plots showing the positive correlation between the Th17 cells (CCR6+CD4+ T cells) vs. MAIT cells in SHIV-infected macaques. (C) Representative Facs plots showing the staining pattern on MR-1 vs. CD161 from five SHIV-infected macaques. (D) Representative Facs plots showing the production of cytokines (IFN-γ, TNF-α, IL-17, IL-22, IFN-γ+TNF-α+, and IL-17+IL-22+ cells) by IL-18R+ and IL-18R-ve MAIT cells during chronic SHIV infection in an animal. (E) IL-18R expression did not show any difference in IFN-γ+ or IL-17+ single positive cytokine (n = 5). [file Image_2.TIFF]
